# Disentangling a Holobiont – Recent Advances and Perspectives in *Nasonia* Wasps

**DOI:** 10.3389/fmicb.2016.01478

**Published:** 2016-09-23

**Authors:** Jessica Dittmer, Edward J. van Opstal, J. Dylan Shropshire, Seth R. Bordenstein, Gregory D. D. Hurst, Robert M. Brucker

**Affiliations:** ^1^Rowland Institute at Harvard, Harvard University, CambridgeMA, USA; ^2^Department of Biological Sciences, Vanderbilt University, NashvilleTN, USA; ^3^Department of Pathology, Microbiology, and Immunology, Vanderbilt University, NashvilleTN, USA; ^4^Institute of Integrative Biology, University of LiverpoolLiverpool, UK

**Keywords:** host-symbiont interactions, microbiome, *Nasonia*, symbiosis, *Wolbachia*, Arsenophonus

## Abstract

The parasitoid wasp genus *Nasonia* (Hymenoptera: Chalcidoidea) is a well-established model organism for insect development, evolutionary genetics, speciation, and symbiosis. The host-microbiota assemblage which constitutes the *Nasonia* holobiont (a host together with all of its associated microbes) consists of viruses, two heritable bacterial symbionts and a bacterial community dominated in abundance by a few taxa in the gut. In the wild, all four *Nasonia* species are systematically infected with the obligate intracellular bacterium *Wolbachia* and can additionally be co-infected with *Arsenophonus nasoniae.* These two reproductive parasites have different transmission modes and host manipulations (cytoplasmic incompatibility vs. male-killing, respectively). Pioneering studies on *Wolbachia* in *Nasonia* demonstrated that closely related *Nasonia* species harbor multiple and mutually incompatible *Wolbachia* strains, resulting in strong symbiont-mediated reproductive barriers that evolved early in the speciation process. Moreover, research on host-symbiont interactions and speciation has recently broadened from its historical focus on heritable symbionts to the entire microbial community. In this context, each *Nasonia* species hosts a distinguishable community of gut bacteria that experiences a temporal succession during host development and members of this bacterial community cause strong hybrid lethality during larval development. In this review, we present the *Nasonia* species complex as a model system to experimentally investigate questions regarding: (i) the impact of different microbes, including (but not limited to) heritable endosymbionts, on the extended phenotype of the holobiont, (ii) the establishment and regulation of a species-specific microbiota, (iii) the role of the microbiota in speciation, and (iv) the resilience and adaptability of the microbiota in wild populations subjected to different environmental pressures. We discuss the potential for easy microbiota manipulations in *Nasonia* as a promising experimental approach to address these fundamental aspects.

## Introduction

Bacterial symbionts are widely recognized as important drivers of insect physiology, development, behavior, reproduction, nutrition, and evolution (Buchner, 1965; Moran, 2007; Douglas, 2010, 2015). Historically, symbiosis research focused primarily on binary interactions between insect hosts and particular symbionts, whether they are harmful or helpful (Duron et al., 2008; Moran et al., 2008; Moya et al., 2008; Werren et al., 2008). The advent of new DNA sequencing technologies over the last 10 years resulted in what has recently been termed ‘the microbiome revolution’ (Blaser, 2014), providing an unprecedented wealth of information on insect microbiotas from various species. Biologists now recognize that symbioses are shaped by complex multipartite interactions, not only between the host and its associated microbes, but also between different members of the microbial community and the environment. This understanding has led to the view of hosts as complex ecosystems (McFall-Ngai et al., 2013; Sicard et al., 2014), and to the recognition that a more holistic approach is needed to understand the role of the microbiota in major facets of host biology (Gilbert et al., 2012). In insects, the microbiota can modulate numerous host phenotypes spanning development (Shin et al., 2011), nutrition (Chandler et al., 2011; He et al., 2013; Wong et al., 2014), immunity (Chu and Mazmanian, 2013), vector competence and susceptibility to pathogen infection (Dong et al., 2009; Koch and Schmid-Hempel, 2012), among others. The microbiota can also mediate reproductive isolation and thus the mechanisms that drive speciation (Brucker and Bordenstein, 2012c, 2013; Shropshire and Bordenstein, 2016), underscoring the need to understand host-microbiota dynamics over evolutionary timescales.

The recognition of the significance and complexity of host-microbiota interactions has led to the revival of old terms and the establishment of new ones to describe host-microbiota assemblages: As such, the term “holobiont”, originally coined by Margulis (1991), is now frequently used to refer to a host together with its entire microbial consortium, while the “hologenome” encompasses the genomes of all members of the holobiont (Rosenberg et al., 2007; Zilber-Rosenberg and Rosenberg, 2008). These terms provide structural definitions that can be universally applied to any host-microbiota assemblage. Moreover, they are pluralistic in that they encompass constant or inconstant, intracellular or extracellular, horizontally or vertically transmitted, harmful or helpful microbial symbionts (Rosenberg et al., 2007; Bordenstein and Theis, 2015; Theis et al., 2016). This perception of a holobiont therefore embraces both competition and cooperation between a host and its associated microbes. This is particularly obvious in the case of symbionts like *Wolbachia*, which override host reproduction to increase their own transmission (Duron et al., 2008; Werren et al., 2008). More generally, the microbial partners present in a host organism contribute to the “extended phenotype” of this particular host-symbiont assemblage, i.e., the holobiont. However, many aspects regarding holobionts need to be elucidated: For instance, one may ask whether phenotypic variation in traits, caused by different holobiont assemblies, could drive a multigenerational response to selection, as originally proposed as part of the hologenome concept of evolution (Rosenberg et al., 2007; Zilber-Rosenberg and Rosenberg, 2008). Moreover, if there is a response to selection, does it occur at the host, microbe, or microbial community level? While the broad utility of the hologenome concept remains debated (Bordenstein and Theis, 2015; Moran and Sloan, 2015; Douglas and Werren, 2016; Theis et al., 2016), it is clear that the microbiome represents an important component of insect biology as well as a source of phenotypic and evolutionary novelty.

With tools available to investigate the diversity and complexity of host-microbe associations, the next challenge will be to disentangle the holobiont in a functional context to understand (i) how different microbes, alone or in synergy, contribute to host phenotype and fitness; (ii) the role of the host, the symbionts and the environment in establishing and regulating the microbiota with each generation; (iii) the role of the microbiota in evolutionary processes such as speciation; and (iv) the resilience and adaptability of the microbiota in wild populations subjected to different environmental pressures.

In this review, we present the parasitoid wasp genus *Nasonia* (Hymenoptera: Chalcidoidea) as an excellent model to experimentally investigate fundamental aspects and evolutionary dynamics of host-microbiota interactions. In particular, we focus on how symbiotic bacteria – both intracellular and the extracellular microbiota – influence *Nasonia* biology, reproduction, and speciation.

## *Nasonia* As A Model Organism

**FIGURE 1:**
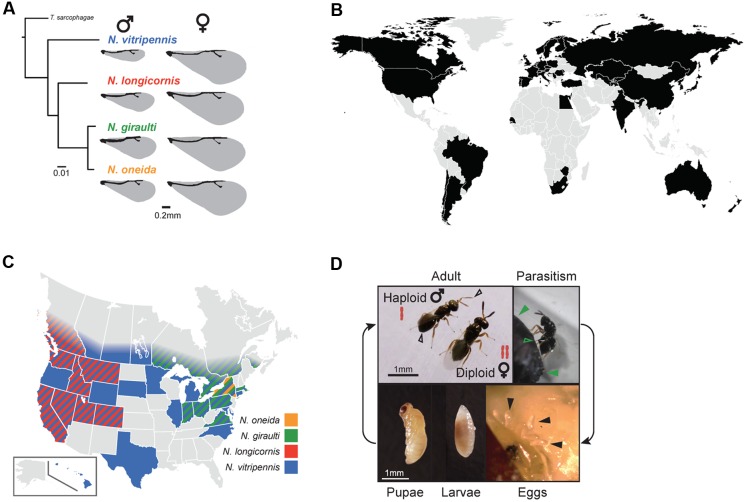
**(A)** Phylogenetic relationships within the *Nasonia* species complex based on the CO1 gene. The parasitoid wasp *Trichomalopsis sarcophagae* was used as outgroup. The scale bar indicates substitutions per site. Note the morphological differences in wing size between species and genders (drawing based on Loehlin et al., 2010). **(B)** Countries in which *Nasonia* has been observed, based on published records of *N. vitripennis* [Universal Chalcidoidea Database (Noyes, 2016) and (Raychoudhury et al., 2009, 2010b; Paolucci et al., 2013)]. The gray color indicates countries for which no observations are documented, in most cases due to missing sampling information. For all countries except the US, records state that the observed species was *Nasonia vitripennis* – however, many of these observations were made before the discovery of the three younger species in the US. Therefore, this global map shows observations of *Nasonia* without distinguishing between species. **(C)** Observations of all four *Nasonia* species in the US and Canada, based on published information (Darling and Werren, 1990; Raychoudhury et al., 2009, 2010a,b). **(D)** *Nasonia* life cycle from oviposition to adulthood. Sex-specific differences in ploidy are indicated for adult males and females. Additional sexual dimorphisms include smaller wing size, less pigmented antennae and rounded abdomen in males (open black arrows). Parasitism is representing by a female wasp ovipositing into a fly pupa (closed green arrows: Fly pupa; open green arrows: Ovipositor of the wasp). Embryos are approximately 100 μm by 500 μm in size (closed black arrows). The *Nasonia* larva and pupa were photographed outside of their fly host. Photo credit: Matthew C. Johnson © 2016

The parasitoid wasp genus *Nasonia* (also referred to as “jewel wasp”) is a species complex comprised of four interfertile species: *N. vitripennis*, *N. longicornis*, *N. giraulti*, and *N. oneida* (**Figure 1A**) (Darling and Werren, 1990; Raychoudhury et al., 2010a). The older species *N. vitripennis* is estimated to have diverged from the three younger species 1 million years ago (mya). The other species were discovered only in the last 26 years and have diverged 0.4 mya in the case of *N. longicornis* and *N. giraulti* and 0.3 mya in the case of *N. giraulti* and *N. oneida* (Werren and Loehlin, 2009; Werren et al., 2010). While *N. vitripennis* is cosmopolitan (**Figure 1B**), the three younger species have only been observed in North America, where they show species-specific distributions: *N. longicornis* is restricted to the west, *N. giraulti* to the northeast and the most recently discovered species *N. oneida* has so far only been observed in New York state (**Figure 1C**) (Darling and Werren, 1990; Raychoudhury et al., 2010a). All species are parasitoids of fly pupae (Werren and Loehlin, 2009; Desjardins et al., 2010). Adult *Nasonia* females lay their eggs within the fly puparium (**Figure 1D**) and inject a venom that prevents the fly from mounting an immune response against the intruders (Danneels et al., 2014). A single fly pupa may be parasitized by multiple females of the same or different species (superparasitism and multiparasitism, respectively) (Darling and Werren, 1990). Under laboratory conditions (constant temperature of 25°C), *Nasonia* has a short generation time of only 14 days: Larvae emerge 24-36 h after egg laying and undergo four larval instars (during which they feed on the fly host), followed by pupation after 7-8 days and emergence from the fly as adults (**Figure 1D**).

*Nasonia* is a well-established model for insect development (Rosenberg et al., 2014), behavior (Bertossa et al., 2013), sex determination (Beukeboom and van de Zande, 2010; Verhulst et al., 2010), evolutionary genetics (Desjardins et al., 2010, 2013; Loehlin et al., 2010), immunity (Tian et al., 2010; Brucker et al., 2012; Sackton et al., 2013), speciation (Breeuwer and Werren, 1995; Ellison et al., 2008; Buellesbach et al., 2013; Gibson et al., 2013) and symbiosis with the reproductive parasites *Wolbachia* (Breeuwer and Werren, 1990; Bordenstein et al., 2001; Raychoudhury et al., 2009) and *Arsenophonus* (Huger et al., 1985; Gherna et al., 1991; Ferree et al., 2008). In addition, *Nasonia* is emerging as a model for insect-gut microbiota symbioses across recent evolutionary time periods and speciation events (Brucker and Bordenstein, 2012b, 2013). Major reasons for this versatility are its ease of rearing in the laboratory, the ability to establish interspecies hybrids after curing of *Wolbachia*, and the advantages of haplodiploid sex determination, wherein males and females develop from unfertilized (haploid) and fertilized (diploid) eggs, respectively (**Figure 1D**). Haplodiploidy is particularly useful for quantitative genetics as all recessive alleles are expressed in the male haploid state (Werren and Loehlin, 2009).

**FIGURE 2:**
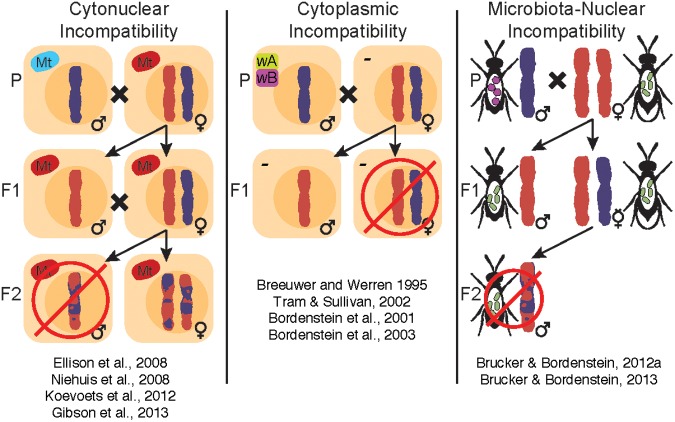
**Within the *Nasonia* clade, there are three published sources of hybrid incompatibilities: cytonuclear, cytoplasmic, and microbiota-nuclear incompatibilities.** Cytonuclear incompatibilities, or negative interactions between mitochondria and the nuclear genome, are associated with lethality in F_2_ males from younger interspecific crosses (*N. giraulti* and *N. longicornis*) and near complete lethality in older interspecific crosses (*N. vitripennis* and *N. giraulti* or *N. longicornis*). Hybrid lethality has some plasticity due to environmental factors (Koevoets et al., 2012), but clear cytonuclear incompatibilities that complicate development and gene regulation (Ellison et al., 2008; Niehuis et al., 2008; Koevoets et al., 2012; Gibson et al., 2013) have been genetically mapped across the *Nasonia* genomes. Cytoplasmic incompatibilities are a consequence of infection with different *Wolbachia* strains (*wA* and *wB*), which causes post-fertilization chromatin defects that result in inviable fertilized eggs (Bordenstein et al., 2001, 2003; Tram and Sullivan, 2002). Finally, microbiota-nuclear incompatibilities result from negative interactions between the microbiota and host genome and lead to hybrid lethality, altered microbial communities and innate immune regulation (Brucker and Bordenstein, 2013). The collective influences of these incompatibilities on *Nasonia* make it a powerful model for evolutionary and symbiotic studies of speciation and reproductive isolation. How these incompatibilities have evolved relative to each other is an important avenue for future research.

Perhaps the most recognizable aspect of *Nasonia* biology is the body of literature on speciation. Several types of hybrid maladies have been studied in *Nasonia*, including cytonuclear, cytoplasmic and microbiota-nuclear incompatibilities (**Figure 2**). Specifically, cytonuclear incompatibilities exist between mitochondrial and nuclear genes presumably involved in the oxidative phosphorylation pathway, leading to reduced energy production and hybrid fitness (Ellison et al., 2008; Niehuis et al., 2008; Koevoets et al., 2012; Gibson et al., 2013). On the other hand, cytoplasmic and microbiota-nuclear incompatibilities result from the influence of bacterial endosymbionts (e.g., *Wolbachia*) or the extracellular microbiota on reproductive fitness, either by affecting offspring viability at the embryonic stage or by altering the immune response of developing larvae, respectively (Bordenstein et al., 2001; Brucker and Bordenstein, 2013). These incompatibilities influence *Nasonia* reproductive isolation in an additive fashion, since removal of one incompatibility does not necessarily remove the others (Brucker and Bordenstein, 2013).

The growing interest in *Nasonia* has also resulted in a wealth of available resources, many of which are advantageous for the study of host-microbe interactions: Annotated genomes are available for all species except *N. oneida* (Werren et al., 2010), together with an extensive genetic toolbox (reviewed in Werren and Loehlin, 2009; Lynch, 2015), transcriptome and methylome for *N. vitripennis* (Sackton et al., 2013; Wang et al., 2013; Beeler et al., 2014), a well-characterized complex innate immune system (Tian et al., 2010; Brucker et al., 2012; Sackton et al., 2013) and a procedure for host genetic manipulation via RNAi (Lynch and Desplan, 2006; Werren et al., 2009). The most promising technique for the purpose of this review is the recently developed *in vitro* rearing technique, allowing the successful rearing of *Nasonia* from embryos to adults outside of its fly host (Brucker and Bordenstein, 2012a; Shropshire et al., 2016), thereby providing the means to establish axenic and gnotobiotic lineages.

## Two Reproductive Parasites With Different Lifestyles

A prominent feature of insect symbioses is that many species entertain long-lasting associations with heritable obligate mutualistic endosymbionts (primary symbionts) (Moran et al., 2008; Moya et al., 2008; Koga et al., 2013). While this type of symbiosis is absent in *Nasonia*, all *Nasonia* species harbor a different type of heritable endosymbiont - reproductive parasites. Instead of conferring an obvious benefit to their host, these bacteria are facultative symbionts that have evolved different strategies to manipulate host reproduction in order to promote their own vertical transmission from mother to offspring (Hurst and Frost, 2015). The most common phenotype in insects is cytoplasmic incompatibility (CI), a reproductive incompatibility between sperm and egg preventing normal mitosis (Serbus et al., 2008). Other reproductive manipulations result in female-biased sex-ratios caused by parthenogenesis, male-killing or the feminization of genetic males (Stouthamer et al., 1993; Hurst et al., 2003; Narita et al., 2007; Bouchon et al., 2008). The common theme of these reproductive manipulations is that they increase the number of infected females in host populations, thereby enhancing maternal symbiont transmission. Bacteria of the genus *Wolbachia* (Alphaproteobacteria) are by far the best-studied and the most frequently encountered reproductive parasites (Hilgenboecker et al., 2008; Werren et al., 2008; Sicard et al., 2014), but other bacteria (*Rickettsia* (Alphaproteobacteria), *Arsenophonus* (Gammaproteobacteria), *Cardinium* and *Flavobacterium* (Bacteroidetes) and *Spiroplasma* (Mollicutes)] are also able to induce at least one reproductive manipulation (Duron et al., 2008; Hurst and Frost, 2015).

In the wild, all four *Nasonia* species are ubiquitously infected (100%) with *Wolbachia* in North America and Eurasia (Bordenstein et al., 2001; van Opijnen et al., 2005; Raychoudhury et al., 2009; Raychoudhury et al., 2010b), while a fraction of *N. vitripennis* and *N. longicornis* females (approximately 5%) additionally carry *Arsenophonus nasoniae* (Gherna et al., 1991; Balas et al., 1996). These symbionts are highly different in terms of reproductive manipulation -*Wolbachia* induces CI, while *Arsenophonus* is a male-killer (historically referred to as the ‘Son-Killer’ in *Nasonia* (Skinner, 1985; Gherna et al., 1991)). The two symbionts also differ in their vertical transmission mechanisms: *Wolbachia* are transmitted transovarially (Breeuwer et al., 1992), while *Arsenophonus* depends on horizontal or environmental transmission and establishes new infections after ingestion (Werren et al., 1986; Gherna et al., 1991; Parratt et al., 2016). Therefore, these associations provide ample opportunities for studying the evolution of different symbiotic lifestyles in bacteria and the role of bacterial endosymbionts on host physiology and evolution, including reproductive processes and speciation.

### *Wolbachia*, An Influential Partner Over Evolutionary Time-Scales

*Wolbachia* are widespread obligate intracellular Alphapro-teobacteria, estimated to infect 40-65% of insect species (Hilgenboecker et al., 2008; Werren et al., 2008; Zug and Hammerstein, 2012). While being primarily maternally transmitted, horizontal transfers have frequently occurred over evolutionary time-scales (O’Neill et al., 1992; Rousset et al., 1992; Werren et al., 1995). To date, *Wolbachia* strains are divided into 16 clades, referred to as “supergroups” A-Q (Lo et al., 2007; Ramírez-Puebla et al., 2015).

The *Nasonia* species complex has been a major model system for *Wolbachia*-insect symbioses for more than 25 years (Breeuwer and Werren, 1990). This young species complex has enabled scientists to reconstruct the history of *Wolbachia* acquisitions and transmission routes across the *Nasonia* clade (van Opijnen et al., 2005; Raychoudhury et al., 2009). Moreover, the ability to produce interspecies hybrids has been exploited to introgress the cytotype (including the *Wolbachia*) of a given species into the nuclear genotype of another, thereby providing insights into *Wolbachia*-host genotype interactions, different modes of CI and the role of *Wolbachia* in speciation (Breeuwer and Werren, 1990, 1993b, Bordenstein and Werren, 1998; Bordenstein et al., 2001, 2003; Chafee et al., 2011; Raychoudhury and Werren, 2012).

**FIGURE 3:**
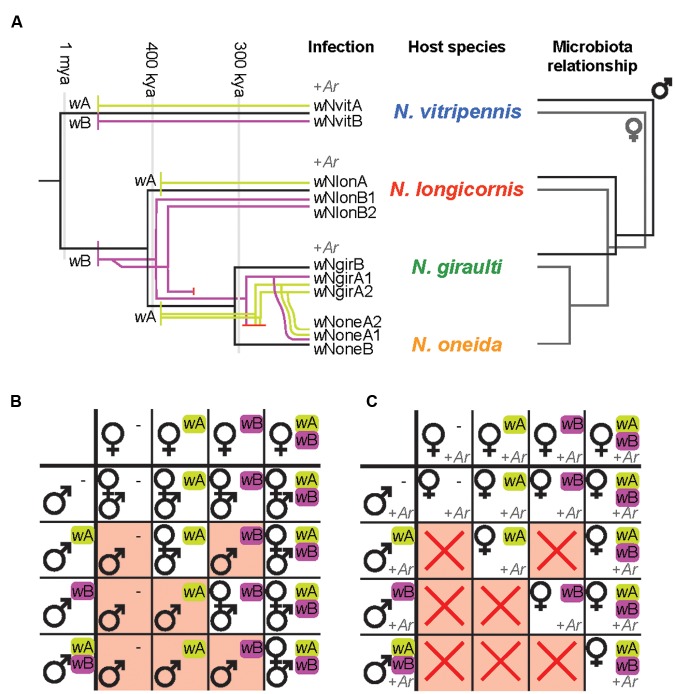
**(A)** *Wolbachia-Nasonia* associations and phylosymbiosis (modified from Raychoudhury et al., 2009; Brucker and Bordenstein, 2012b,c). *Wolbachia* acquisitions and subsequent divergence are overlaid on the *Nasonia* phylogeny. Strains from *Wolbachia* supergroup A are represented in green, strains from supergroup B in purple. *Arsenophonus* (+*Ar*) has been found to infect three species of *Nasonia*. The microbial community relationships parallel the host phylogeny, indicating species-specific microbiota assemblies that establish phylosymbiosis. This pattern has been observed in males for three species (Brucker and Bordenstein, 2012b, 2013) as well as in females for all four species (R. M. Brucker and S. R. Bordenstein, personal communication). **(B)** Impact of *Wolbachia*-induced CI on offspring production. *Wolbachia* present in males induce a sperm modification that needs to be rescued by the same *Wolbachia* strain in the fertilized egg for normal offspring production. CI (red background) occurs if the female is uninfected (-) (unidirectional CI) or harbors a different *Wolbachia* strain (bidirectional CI) and results in male-only (or male-biased) broods due to loss of the paternal chromosomes. Note that although *Wolbachia* modify male sperm, the symbiont is only maternally transmitted. Offspring will therefore harbor the same *Wolbachia* strain(s) as their mothers. *w*A/*w*B indicate different *Wolbachia* strains. **(C)** Impact of *Arsenophonus*-induced male-killing on offspring production, in combination with *Wolbachia*-mediated CI. Male-killing results in all-female broods in the absence of CI (white background) and no offspring production in combination with CI (red crosses), since the males that are not affected by CI would be killed by male-killing.

The emerging picture of the *Nasonia-Wolbachia* association is as follows: The four *Nasonia* species together harbor 11 different *Wolbachia* strains from the A and B supergroups (**Figure 3A**) (Raychoudhury et al., 2009), two major arthropod-*Wolbachia* clades that diverged about 60 million years ago (Werren et al., 1995). *N. vitripennis* carries two *Wolbachia* strains (one from each supergroup), while the three younger species are all triple infected: *N. giraulti* and *N. oneida* both harbor two supergroup A strains and one supergroup B strain, whereas *N. longicornis* harbors one supergroup A strain and two supergroup B strains (**Figure 3A**) (Raychoudhury et al., 2009). Comparing the phylogenetic relationships between these *Wolbachia* strains with host phylogenies based on nuclear and mitochondrial genes revealed that several *Wolbachia* strains were most likely acquired independently via horizontal transfers from other insects, including *Drosophila* spp. (supergroup A strains of *N. giraulti* and *N. longicornis*), the parasitoid wasp *Muscidifurax uniraptor* (supergroup A strain of *N. vitripennis*) as well as the blowfly *Protocalliphora sialia* (supergroup B strain of *N. vitripennis*) (van Opijnen et al., 2005; Raychoudhury et al., 2009). The latter cases point towards an ecological interaction as the source of the *Wolbachia* transfers to *N. vitripennis*, since both *Nasonia* and *M. uniraptor* parasitize blowflies. Indeed, horizontal transfers between parasitoids and their fly hosts as well as between different parasitoid species infecting the same host are known to occur occasionally (Heath et al., 1999; Vavre et al., 1999; Huigens et al., 2004).

The supergroup B *Wolbachia* of *N. longicornis* and *N. giraulti* were acquired prior to the speciation of the two species and subsequently co-diverged with their hosts (**Figure 3A**) (van Opijnen et al., 2005; Raychoudhury et al., 2009). Hence, the supergroup B strain from *N. giraulti* is nearly identical to one of the B strains from *N. longicornis* and the estimated divergence time of the two *Wolbachia* strains coincides with the divergence of their host species, i.e., about 0.4-0.5 mya (van Opijnen et al., 2005; Raychoudhury et al., 2009). In addition, the second B strain of *N. longicornis* is estimated to have diverged from the other B strains about 1.5 mya. Considering that this time point was long before the speciation of the two host species, it is likely that the common ancestor of *N. longicornis* and *N. giraulti* harbored two B strains, one of which was lost in *N. giraulti* after the speciation event (Raychoudhury et al., 2009). A similar co-divergence event between the A strains of *N. longicornis* and *N. giraulti* is possible, but the similarity of these strains with *Wolbachia* from several *Drosophila* species currently makes it impossible to rule out independent horizontal transfer events (Raychoudhury et al., 2009). The three *Wolbachia* strains in the recently discovered species *N. oneida* are identical (for 5 house-keeping genes and the *wsp* gene) to those of the closely related *N. giraulti* and have likely been acquired via hybridisation between the two species, resulting in a mitochondrial-*Wolbachia* sweep from *N. giraulti* to *N. oneida* (**Figure 3A**) (Raychoudhury et al., 2009). Future phylogenomic comparisons based on the entire genomes of the different *Wolbachia* strains will be needed to obtain a higher resolution. Nonetheless, these findings illustrate a high *Wolbachia* diversity in the *Nasonia* species complex, along with various patterns of *Wolbachia* transfers within a single insect genus.

One of the most prominent aspects of *Wolbachia* is undoubtedly its ability to manipulate host reproduction in various ways (Werren et al., 2008). All *Nasonia*-associated *Wolbachia* induce CI, a reproductive incompatibility consisting of two components: A symbiont-induced modification of the paternal chromosomes during spermatogenesis that needs to be ‘rescued’ by the same symbiont being present in the fertilized egg (Tram and Sullivan, 2002; Serbus et al., 2008). If the female is uninfected (unidirectional CI) or harbors a different bacterial strain (bidirectional CI), the modification may not be rescued, resulting in CI (**Figure 3B**). Consequently, infected females have a fitness advantage over uninfected females, since they can reproduce successfully with all available males, regardless of male infection status. Similarly, bidirectional CI results in a fitness benefit for multiply infected females since they are at lower risk to suffer from CI (Mouton et al., 2003, 2004). The fact that all *Nasonia* species generally harbor double or triple infections that are mutually incompatible reinforces bidirectional incompatibility between all species pairs, with the notable exception of *N. giraulti* and *N. oneida*, whose *Wolbachia* strains are identical (Breeuwer and Werren, 1990; Breeuwer et al., 1992; Bordenstein and Werren, 1998, 2007; Bordenstein et al., 2001, 2003; Raychoudhury et al., 2010a). While the exact molecular mechanisms of CI are still not understood, it is evident that the paternal chromosomes fail to condense correctly and may be lost during the first mitotic division, causing embryo mortality in diploid organisms (Tram and Sullivan, 2002). In contrast, in haplodiploid insects such as *Nasonia*, CI can be manifested in different ways. The most extreme phenotypes are Male Development and Female Mortality (Vavre et al., 2000; Bordenstein et al., 2003; Vavre et al., 2009). In the first case, the paternal genome is completely lost, which restores haploidy in fertilized eggs and results in the conversion of female into male offspring. Hence, this type of CI is characterized by all-male broods (**Figure 3B**) with little or no embryonic mortality compared to compatible crosses. In contrast, an incomplete loss of the paternal chromosomes would instead cause aneuploidy and a high mortality of fertilized eggs (i.e., female offspring), resulting in smaller broods with male-biased sex-ratios (Bordenstein et al., 2003). It has been hypothesized that paternal chromosome loss was mediated by the intensity of *Wolbachia*-induced sperm modification, with less efficient modifications leading to only partial chromosome loss and aneuploidy in fertilized eggs (Vavre et al., 2009). While CI-induced mortality seems to be common in haplodiploid species, including the younger *Nasonia* species *N. longicornis* and *N. giraulti* (Bordenstein et al., 2003; Dedeine et al., 2004; Vavre et al., 2009), *N. vitripennis* is an exception from the rule in that the Male Development type is the predominant CI phenotype in this species (Breeuwer and Werren, 1990; Bordenstein et al., 2003). Interestingly, interspecies crosses and introgression experiments between *N. vitripennis* and *N. giraulti* revealed that this rare CI phenotype is determined by the *N. vitripennis* nuclear genotype rather than *Wolbachia*-related effects, since it is even observed in incompatible crosses between infected *N. giraulti* males and uninfected *N. vitripennis* females (Bordenstein et al., 2003). These results show that the host genotype may determine the fate of the paternal chromosomes in fertilized eggs, independent of *Wolbachia* (Bordenstein et al., 2003). On an evolutionary scale, the conversion of incompatible fertilized eggs into viable haploid males may represent a selective advantage for the more widely distributed *N. vitripennis*, in that it prevents embryo mortality as a consequence of incompatible matings with its microsympatric sister species (Bordenstein et al., 2003). Taken together, the above illustrates the preeminent role of *Nasonia* as a model to understand host-*Wolbachia* interactions, notably in terms of diverse acquisition routes, *Wolbachia*-host genotype interactions and modes of CI.

### *Arsenophonus*, the Son-Killer

The genus *Arsenophonus* lies in the Gammaproteobacteria and is most closely related to the genera *Proteus*, *Providencia*, and *Photorhabdus*. Members of this genus establish diverse symbiotic interactions with insect hosts, with around 5% of insect species estimated to carry the symbiont (Duron et al., 2008). Many, but not all interactions are based on heritable symbiosis – passage from female to her progeny. However, unlike many heritable symbionts (e.g., *Wolbachia*), there is substantial diversity in the transmission biology of this microbe. Some strains maintain substantial ‘infectious’ transmissions through the environment, others show vertical transmission via maternal transfer of the microbe to the egg surface followed by ingestion of the symbiont by the hatching larva, and others show classical maternal inheritance through the egg cytoplasm (see **Table 1** for the diversity of *Arsenophonus*-host interactions).

**Table 1:** Diversity of *Arsenophonus* interactions with insects as exemplified by case studies.

*Arsenophonus* strain	Host species	Impact on insect host	Transmission biology	Reference
*Ca.* Riesia pediculola	Louse	Required for host function; supplies B vitamins	Bacteriome symbiont with maternal inheritance through eggs	Kirkness et al., 2010
*Ca.* Arsenophonus melophagi	Hippoboscid flies	Required for host function; supplies B vitamins	Bacteriome symbiont with maternal inheritance through milk gland	Novakova et al., 2015
*Ca.* Arsenophonus triatominarum	Triatomine bugs	Not required by host; impact on host unclear	Maternal inheritance inside eggs	Hypsa and Dale, 1997
*Arsenophonus nasoniae*	*Nasonia* wasps	Son-killer; sons of infected females die during early embryogenesis	Maternal inheritance via calyx fluid upon oviposition followed by *per os* uptake; also infectious transmission during superparasitism	See main text
*Ca.* Phlomobacter frageriae	Cixiid bugs	Unknown	Passed to, and acquired from, plant hosts during feeding. Vertical transmission with low efficiency	Bressan, 2014
*Arsenophonus* sp.	Aphids	Positive impact on fitness in assays, unknown mechanism (not protective)	Vertical transmission through eggs	Wulff and White, 2015


*Arsenophonus nasoniae*, the symbiont of *Nasonia* wasps, represents the type species of the genus (Gherna et al., 1991). The discovery of the symbiont followed observations of maternally inherited sex ratio biases in certain *N. vitripennis* isofemale lines. These lineages were typified by the presence of all (or near all) female broods associated with the death of male progeny, referred to as the ‘son-killer’ trait (**Figure 3C**) (Skinner, 1985). The trait was heritable through the female line. Subsequent microscopical examination recorded diffuse microbial infections throughout the soma of affected females (Huger et al., 1985). Unusual for heritable microbes, the infection was relatively easily isolated into pure culture, and reinjection into fly pupae alongside ovipositing *Nasonia* led to establishment of the son-killer trait, fulfilling Koch’s postulates for this microbe (Werren et al., 1986). Later, male-killing was observed to be confined to unfertilized (rather than simply haploid) eggs, and associated with destruction of the maternal centrosome upon which unfertilized eggs depend for development, while diploids, which also inherit a paternal centrosome, do not (Ferree et al., 2008).

The early experiments of Skinner (1985) demonstrated that unlike other heritable microbes known at the time, *A. nasoniae* possessed the capacity for infectious transmission in addition to maternal inheritance. He observed that when an infected and uninfected female used the same host fly pupa (superparasitism), the progeny from the uninfected female acquired the infection, which would then be passed on in turn by them to their progeny. The infectious transmission is a result of the microbe not being inherited within eggs, but passed through calyx fluid deposited in the fly pupa during oviposition. The microbe grows saprophytically inside the deceased fly puparium, and is ingested by the developing *Nasonia* larvae, whether they are the progeny of the infected female or are derived from co-parasitising individuals. Following ingestion, the microbe enters the wasp through the gut epithelium to produce the diffuse infection seen in the adult soma. This infection includes presence in the calyx gland, thus ensuring *A. nasoniae* onward transmission. *Arsenophonus* thus has a saprophytic stage, a stage where it is a component of the gut microbiota, and a systemic infection stage.

This unusual life cycle is distinct from many other heritable microbe-host interactions and has important biological consequences in terms of the population dynamics of *A. nasoniae* infections in natural populations. First, superparasitism (and the infectious transmission that follows from it) apparently represents a necessary condition for the maintenance of the microbe (Parratt et al., 2016). In laboratory emulation, *A. nasoniae* was lost from wasp populations where females were forced to oviposit alone, because vertical transmission is leaky (10% of progeny do not inherit the microbe). In contrast, where females are forced to oviposit in patches with other females and superparasitism rates are high, the infection spreads to fixation in just 3-5 generations. In experiments where the opportunity for superparasitism varies between these global absence/presence extremes, the prevalence achieved is a function of the superparasitism opportunity. Thus, in contrast to other heritable reproductive parasitic microbes, infectious transmission is necessary for *A. nasoniae* maintenance, and son-killing appears to represent a secondary benefit to the microbe.

A likely consequence of this transmission biology is also the presence of multiple *A. nasoniae* strains within an individual. The presence of co-infections is likely to erode the correlation between host and microbe fitness that selects for benign and beneficial infections, as the ability to compete (and outcompete) other strains is also a fitness-related trait for the microbe. In this context, it is notable that the genome carries colicin elements whose canonical function is to disable cells that do not carry the element (Wilkes et al., 2010). Further, *A. nasoniae* represents an antagonist of *Nasonia* (male-killing makes the element parasitic) that may drive host evolution to prevent its acquisition; as such it may be a driver of immune system evolution that may impact upon other microbiota members.

The transmission pathway also has an important consequence for the movement of the symbiont within chalcid wasp communities. (Duron et al., 2010) noted that uninfected individuals of a different species acquired infection during multiparasitism with an *A. nasoniae* infected *N. vitripennis*. This exposure resulted in the efficient transfer of the symbiont into the second species with efficiency comparable to that seen during superparasitism. Using ecological data on multiparasitism rates, the authors concluded that *A. nasoniae* in a female *N. vitripennis* had a 12% chance of transfer to *N. giraulti* in nature. Further to this, the ability of *A. nasoniae* to be maintained in different species of chalcid wasps in the laboratory was (as above) related to their tendency to superparasitize, with *A. nasoniae* being lost in species where females were reluctant to utilize already parasitized pupae (Parratt et al., 2016). These results collectively indicate this heritable microbe passes readily between species through multiparasitism, and may pass freely amongst sympatric *Nasonia* species. Closely related *A. nasoniae* strains have been retrieved from other chalcid parasitoids (Taylor et al., 2011; Bohacsova et al., 2016), indicating this symbiont infects a wide range of parasitic wasps.

Aside from these biological consequences, the unusual biology of *A. nasoniae* makes the system more manipulable than most heritable microbe-host interactions. The saprophytic life style stage is almost certainly the reason this microbe grows readily in cell free culture, a property distinct from other heritable microbes (Werren et al., 1986). Further, the strains can easily be reintroduced into the wasp by injection into the host pupa allowing targeted and controlled study of the interaction of the microbe with different wasp genotypes and other elements of the microbiome. Finally, the microbe has intact systems for recombination, and is likely to be genetically manipulable, which would make this one of the few heritable microbes in which functional genetics, both forward and reverse, are possible. However, this ability comes with the caveat that the microbe is not necessarily a reflective model for heritable symbioses in general – it is genetically and biologically more similar to pathogens and gut ‘commensals’ than the classically considered heritable microbiota.

### Meeting in the Same Host: Multipartite Interactions between Reproductive Parasites

The previous sections have illustrated our growing knowledge regarding binary interactions of *Nasonia* with either *Wolbachia* or *Arsenophonus*. This leads us to an as yet unexplored question: Do the two reproductive parasites also interact with each other in the same host? And if so, what is the outcome? The effects of co-infections studied to date vary immensely. For instance, *Wolbachia* and *Asaia* occupy different niches when co-infecting mosquitoes (Hughes et al., 2014; Rossi et al., 2015). In contrast, male-killing *Spiroplasma* (Mollicutes) have been observed to negatively affect *Wolbachia* densities when co-infecting *D. melanogaster*, while *Wolbachia* had no impact on *Spiroplasma* (Goto et al., 2006). Phenotypically, *Wolbachia* has been shown to interfere with *Cardinium*-induced CI in the spider mite *Bryobia sarothamni*, although little is known about the underlying mechanisms (Ros and Breeuwer, 2009). If *Arsenophonus* also had a negative impact on *Wolbachia*, co-infection could directly affect CI (and thereby population dynamics) in *Nasonia*, since the strength of CI is dependent on *Wolbachia* density (Breeuwer and Werren, 1993a). How *Arsenophonus* and *Wolbachia* interact remains to be determined, but the symbionts may well represent important drivers of each other’s biology.

The co-existence of different symbionts in the same host environment also creates conditions that are permissive for the exchange of genetic information between symbionts. Hence, the detection of a lateral gene transfer from *Wolbachia* to the genome of the *A. nasoniae* strain infecting *N. vitripennis* (Darby et al., 2010) is interesting in several ways: First, its presence indicates that cross-talk between the two symbionts can occur. Second, the transferred gene is highly similar to a *Wolbachia* surface protein and likely has a functional role at the host-symbiont interface (Darby et al., 2010). Bacteriophages associated with either bacterium could be potential agents for lateral gene transfers between these two symbionts (and potentially other members of the *Nasonia* microbiome). Indeed, both *Wolbachia* and *Arsenophonus* are known to have associated phages, a rare feature in bacterial endosymbionts and a potential source of genomic innovation mediating the adaptive plasticity of both symbionts (Kent and Bordenstein, 2010; Duron, 2014). Bacteriophage WO is extremely widespread in arthropod-*Wolbachia* (Masui et al., 2000; Bordenstein and Wernegreen, 2004; Wu et al., 2004; Braquart-Varnier et al., 2005; Gavotte et al., 2007) and renowned for its ability to jump between different *Wolbachia* strains, especially when infecting the same host (Gavotte et al., 2004; Gavotte et al., 2007; Chafee et al., 2010; Kent et al., 2011). This has led to the “intracellular arena hypothesis” whereby genetic material can be exchanged between different bacterial endosymbionts co-occurring in the same intracellular environment (Bordenstein and Wernegreen, 2004). Indeed, the discovery of a *Wolbachia* gene in the genome of *Arsenophonus* (Darby et al., 2010), a prophage-flanked region of the *w*Mel genome on a plasmid of a *Rickettsia* strain infecting the tick *Ixodes scapularis* (Ishmael et al., 2009), and numerous lateral gene transfers from other bacteria associated with phage regions in the *w*Bol1 genome (Duplouy et al., 2013) strongly indicate that WO phages may also vehicle genetic material between *Wolbachia* and other bacterial taxa. Moreover, sequence similarities between phage WO and eukaryotic genes suggest a history of lateral genetic transfers between the two entities (Bordenstein and Bordenstein, 2016). Interestingly, active lytic phages might also be implicated in *Wolbachia* regulation and reduce the strength of CI via a phage-mediated reduction of *Wolbachia* titers (Bordenstein et al., 2006; Bordenstein and Bordenstein, 2011). Regarding *Arsenophonus*, it has recently been shown that the majority of strains harbor the phage APSE (Duron, 2014), previously known from the protective aphid secondary symbiont *Hamiltonella defensa* (Oliver et al., 2003, 2009), suggesting a transfer of phage elements between *Arsenophonus* and *Hamiltonella*. Such an exchange would be all the more relevant as this phage encodes toxicity genes mediating defense against natural enemies of aphids, which happen to be – parasitoid wasps (Oliver et al., 2009). The role of these genes in *Arsenophonus*, a symbiont of parasitoid wasps, remains to be elucidated. Taken together, this section illustrates the complexity and diversity of potential interactions between only two bacterial symbionts infecting the same host organism, without even considering the complexity of the microbiome as a whole.

## Gaining in Complexity: The *Nasonia* Microbiota

Tremendous progress has been made regarding our understanding of the intricate relationships between diverse insects and their co-evolved primary symbionts, particularly regarding metabolic complementarity and metabolite exchange between different partners (McCutcheon et al., 2009; McCutcheon and Moran, 2010; Wilson et al., 2010; Hansen and Moran, 2011; McCutcheon and von Dohlen, 2011; Husnik et al., 2013; Luan et al., 2015) and adaptations of the host immune system to recognize and regulate resident symbionts (Wang et al., 2009; Login et al., 2011; Futahashi et al., 2013; Kim et al., 2013; Shigenobu and Stern, 2013; Masson et al., 2015). However, achieving the same level of insight into host-symbiont cross-talk for highly complex insect microbiotas remains challenging. Many host-associated microbes may not be culturable and therefore impossible to manipulate outside of the host’s body. Hence, we need a study system where the host (i) is easy to rear in the lab; (ii) genetically tractable with resources available for genomic/transcriptomic or immunity-related investigations; (iii) has a complex but well-characterized microbiota; and (iv) this microbiota can be relatively easily manipulated in the host organism, which can be an asset for testing the influence of the microbiome on host traits. Previous work demonstrates that *Nasonia* maintains a relatively high level of microbial diversity, microbiome functionality, and experimental tractability, even while kept under laboratory conditions.

### Microbiota Diversity

**FIGURE 4:**
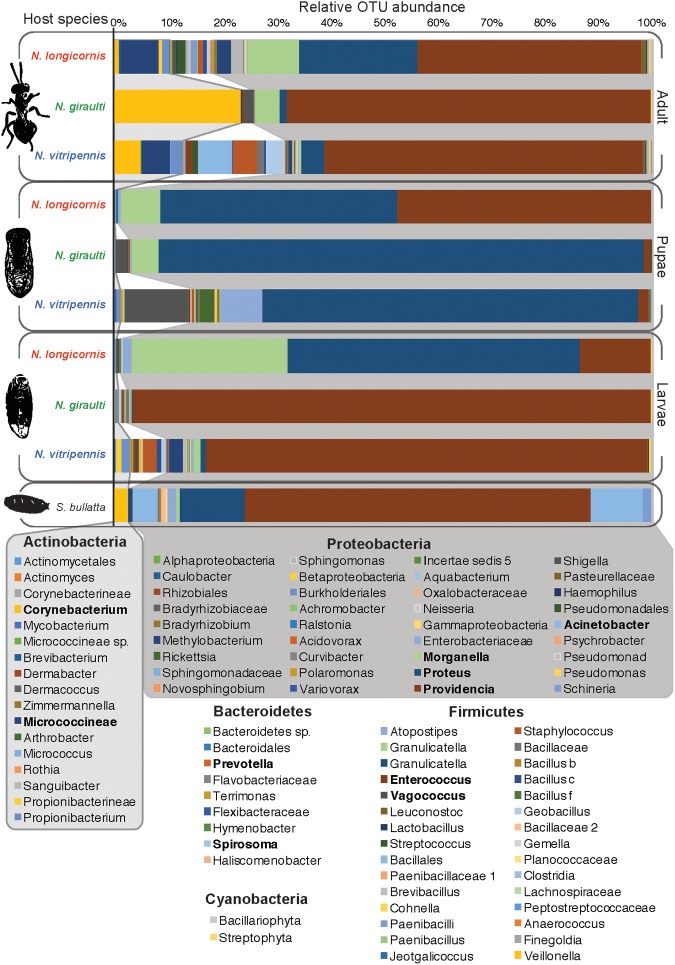
**The relative abundance of bacterial OTUs observed in male *Nasonia* throughout development (Brucker and Bordenstein, 2013).** The OTUs represented across the three *Nasonia* species and their *S. bullata* fly host are dominated by Actinobacteria, Firmicutes, and especially Proteobacteria. Three genera, *Providencia, Proteus*, and *Morganella*, are particularly dominant across all samples. However, their relative abundances differ according to host species and developmental stage. The unparasitized *S. bullata* fly host is similarly dominated by Proteobacteria, specifically the genus *Providencia*. Emboldened OTUs are observed at higher frequencies in one or more samples. It is important to note that many of the rarer OTUs have also been observed in other studies (Brucker and Bordenstein, 2012b and personal communication).

The bacterial diversity of *Nasonia* has been described in lab-reared larvae, pupae and adult males for the three *Nasonia* species *N. vitripennis*, *N. giraulti*, and *N. longicornis* (**Figure 3**) (Brucker and Bordenstein, 2012b, 2013). Microbial diversity in these strains ranged from 44 to 83 OTUs at a 97% identity cutoff and varies between host species and developmental stages (**Figure 4**). Overall bacterial diversity in *Nasonia* is similar to other Hymenoptera, such as honey bees (*Apis mellifera*, 82-116 OTUs), bumblebees (*Bombus* sp., 33-47 OTUs), and fungus farming ants (*Mycocepurus smithii*, an average of 52 OTUs) (Martinson et al., 2011; Cariveau et al., 2014; Corby-Harris et al., 2014; Kellner et al., 2015). Like most insects, the *Nasonia* microbiota is dominated by members of the Proteobacteria phylum. The average *Nasonia* microbiota in adult males is composed of 74.4% Proteobacteria, 15.7% Actinobacteria, and 9.5% Firmicutes (**Figure 4**) (Brucker and Bordenstein, 2013). Interestingly, at the bacterial genus level, there are three major taxa (Gammaproteobacteria) that account for up to 75% of the male microbiota: *Providencia*, *Proteus*, and *Morganella* (**Figure 4**). Alone, *Providencia* sp. compose 59, 68, and 41% of the microbiota in *N. vitripennis*, *N. giraulti*, and *N. longicornis*, respectively. Comparatively, *Nasonia* is a more tractable laboratory model for controlled experiments and is consistently comprised of 4-5 OTUs that make up the majority of all bacterial sequences.

The two genera *Providencia* and *Proteus* are often the most dominant OTUs observed in the three wasp species throughout their development. These same two OTUs are frequently found in the fly host as well, which could represent a natural reservoir of the bacteria for *Nasonia*. Notably, *Nasonia* undergoes bacterial community successions throughout its development: The microbial community remains relatively simple when the developing larvae are feeding on the likewise relatively simple microbiota of the fly pupa. Then, microbiota composition shifts during pupation, a time when the wasp is no longer feeding, and again before emergence as adult wasps (**Figure 4**). As such, *Providencia* and *Proteus* represent 95-100% of the microbiota in larvae (Brucker and Bordenstein, 2012b, 2013). Although the microbiota of pupae is less diverse than the microbiota of adults, both tend to exhibit a reduction in the dominance of Proteobacteria (Brucker and Bordenstein, 2012b, 2013).

While little is known about the specific functional roles of the microbiota in *Nasonia*, several of the major bacterial genera have been previously studied in other insect models. For instance, *Proteus* has been shown to control the gut microenvironment in blowflies from overgrowth by other bacteria (Erdmann et al., 1984). This colonization resistance could be important for *Nasonia*, which feed on a decaying pupal fly host. Another major taxon, *Providencia*, has been implicated in two symbiotic roles: (i) Providing vitamin B to the blood-feeding leech *Haementeria officinali*s (Manzano-Marin et al., 2015) and (ii) acting as a natural control against the insect pathogen *Paenibacillus* in the Japanese honeybee *Apis cerana japonica* (Yoshiyama and Kimura, 2009).

Ongoing studies are now testing the functional significance of the microbiota in different species of *Nasonia* to determine their role in host development, e.g., through immune regulation, nutrition, and other mechanisms. In addition, transplantations of *Nasonia* microbiotas between host species will elucidate whether interspecific microbiotas alter host development traits such as larval size, larval and pupal development time or adult viability in comparison to intraspecific microbiotas. In this context, studies in *D. melanogaster* have demonstrated that axenic individuals suffer from developmental defects along with smaller body size and an altered nutrient metabolism (Shin et al., 2011; Newell and Douglas, 2014). These defects can be rescued by the acetic acid bacterium *Acetobacter pomorum*, which promotes larval growth and reduces lipid and sugar levels by modulating insulin signaling (Shin et al., 2011). In addition, *Lactobacillus plantarum* promotes larval growth in conditions of nutrient scarcity by enhancing protein assimilation and TOR-dependent hormonal growth signals (Storelli et al., 2011). In turn, the *Drosophila* innate immune response is fine-tuned to maintain gut microbiota homeostasis and responds to bacterial pathogens via ROS-production triggered by bacteria-derived uracil, which is released by various opportunistic pathogens but not autochthonous gut microbes (Ryu et al., 2008; Lee et al., 2013).

The microbial community is not limited to bacteria. *Nasonia* also harbors a diverse set of viruses (Bordenstein and Bordenstein, personal communication) and fungi, and their functional effects on the holobiont await further investigation. While no studies to date have investigated *Nasonia’s* fungal microbiota, the original draft of the *Nasonia* transcriptome revealed three novel single-stranded RNA viruses: NvitV-1, -2, -3 (Oliveira et al., 2010). These viruses were not previously found in other insect hosts, though they are related to the *Picornavirales*—a known order of insect pathogens. The observation of novel viruses in the system is interesting from the perspective that viruses are known to influence the biology of other parasitoid wasps. For instance, the virus *Leptopilina boulardi* Filamentous Virus (LbFV) manipulates the foraging behavior of its solitary parasitoid wasp host, *Leptopilina boulardi*, by inducing superparasitism (Varaldi et al., 2003, 2005, 2006). The virus is injected into the fly host together with the parasitoid eggs, allowing it to spread horizontally to uninfected individuals. In contrast to this infectious virus, polydnavirus-like particles have been integrated into the genomes of braconid and ichneumonid wasps and encode particles that contain wasp DNA and proteins which, when injected into the host with the parasitoids’ eggs, enable evasion or direct suppression of the host’s immune response against the parasitoid, thereby contributing significantly to parasitoid fitness (reviewed in Federici and Bigot, 2003).

### Establishment and Transmission of the Microbiota

The changes in the bacterial community throughout development raise questions as to how the *Nasonia* microbial community assembles through metamorphosis. The answer is not yet clear in any animal system but the patterns exhibited by *Nasonia* offer an opportunity to better understand how animals change developmentally and anatomically with their microbiota. Since *Nasonia* embryos are directly deposited within fly host pupae via oviposition, both maternal and fly host microbes could contribute to the initial microbiota assembly of *Nasonia* larvae. Based on the transmission of microbes in *Drosophila* (Bakula, 1969), it is possible that *Nasonia* acquire their first non-endosymbiotic bacteria through ingestion of the chorion during hatching. Alternatively, the microbial community could be passaged via maternal deposition of calyx fluid and venom – using the same process of transmission as *Arsenophonus* (Huger et al., 1985; Werren et al., 1986). During this event, rare microbes could be introduced into the *Nasonia* microbiota. Subsequent colonization of the microbiota would then occur through feeding on the fly host. However, the excretion of the larval gut content prior to pupation presents a marked bottleneck for the microbiota. As larvae and pupae develop, it is possible that *Nasonia* species-specific innate immune genes regulate this community, which would parallel species-specific antimicrobial regulation of the microbiota in *Hydra* (Franzenburg et al., 2013). On the other hand, the innate immune response of honey bees has been shown to be strongly reduced during the pupal stage compared to larvae and adults (Gatschenberger et al., 2013). If this pattern is consistent across the order Hymenoptera, then a weaker immune regulation during the pupal stage could be influential in the mechanisms that establish the new host species-specific microbiota.

An important aspect that is often overlooked is that microbiota composition may not be regulated solely by host mechanisms, but also through interactions between the microbes themselves. From the microbial perspective, a host organism represents an ecosystem consisting of different microhabitats (i.e., niches) (Sicard et al., 2014), and microbes can be expected to differ in their preference for particular niches. Given that the *Nasonia* microbiota consists at least partly of bacteria acquired from its fly host during larval development, one might ask whether the transfer to *Nasonia* as a new host results in fitness consequences for the microbes. While some might be opportunistic and able to find suitable niches, *Nasonia* may represent a dead-end for other microbes, either due to host factors or competition with other bacteria. The latter may be due to competition for a shared resource/niche and/or by direct interference, for instance via the production of bacteriocidal toxins. Moreover, there may be indirect interactions, mediated through host mechanisms. A particular bacterium may, for instance, activate or suppress the host innate immune system, which then affects the proliferation of other bacteria.

An as yet unexplored but highly relevant aspect is the role of heritable symbionts in the establishment and composition of the *Nasonia* microbiota. *Wolbachia*, for instance, are generally highly abundant in various host tissues, thereby limiting available niches and resources for other bacteria (Dittmer et al., 2014, 2016). It is also known to influence other aspects of its host environment, such as immunity, apoptosis and iron homeostasis (Braquart-Varnier et al., 2008; Brownlie et al., 2009; Kremer et al., 2009, 2012; Kambris et al., 2010; Pan et al., 2012). Considering that *Wolbachia* is ubiquitous in *Nasonia* under natural conditions, *Wolbachia* infection represents the natural infection status of *Nasonia*. *Arsenophonus*, on the other hand, is a more variable heritable symbiont in this system and has the ability to efficiently infect uninfected individuals via horizontal/environmental transmission within the same fly host (Duron et al., 2010; Parratt et al., 2016). Investigating the impact of *Arsenophonus* infection on establishment and composition of the wider *Nasonia* microbiota therefore constitutes a promising line of future research.

### Phylosymbiosis

Microbiota composition is shaped by both host and environmental factors (e.g., immunity and diet, respectively (Ley et al., 2008; Ochman et al., 2010; Chandler et al., 2011; Colman et al., 2012; Ridley et al., 2012). While the first can be considered deterministic, the latter would be rather stochastic. However, it can be challenging to disentangle these two components and to precisely determine the relative roles of the host versus the environment on the establishment of a species’ microbial community. Controlled conditions can provide evidence for host-microbiota interactions by removing confounding variations in diet, age and gender, for instance. Indeed, under a controlled experimental design, three *Nasonia* species were found to harbor distinguishable microbiotas whose beta-diversity relationships parallel host phylogeny at all developmental stages (**Figure 3A**) (Brucker and Bordenstein, 2012b,c). The congruence of host phylogeny and dendrograms reflecting relationships in microbiota composition has since been dubbed “phylosymbiosis” (Brucker and Bordenstein, 2013). For a particular set of closely related species, phylosymbiosis predicts that intraspecific microbial communities are more similar than interspecific communities (Bordenstein and Theis, 2015). Based on that, one could hypothesize that (i) microbiota-based models should predict host species origin with high accuracy, and (ii) various topological congruence analyses of host phylogeny and microbiota dendrograms will reveal significant degrees of phylosymbiosis. Furthermore, if phylosymbiosis were driven by both evolutionary and ecological forces, we might also observe that experimental transplants of autochthonous (intraspecific) versus allochthonous (interspecific) microbiota will drive reductions in host survival and fitness. In addition to *Nasonia*, the pattern of phylosymbiosis is evident in *Hominidae* (Ochman et al., 2010; Moeller et al., 2016), *Hydra* (Fraune and Bosch, 2007; Franzenburg et al., 2013), sponges (Easson and Thacker, 2014), ants (Sanders et al., 2014), and bats (Phillips et al., 2012). One future area of investigation will be to understand the factors influencing phylosymbiosis, e.g., fine-tuned host immune mechanisms and/or different transmission modes (i.e., through maternal transmission or environmental acquisition).

### The Microbiota and Reproductive Isolation

Our growing knowledge of the many ways in which microbial symbionts can induce changes in host phenotypic traits raises the question - to what extent do the microbiota contribute to host diversification, reproductive isolation barriers, and speciation (see Brucker and Bordenstein, 2012c; Vavre and Kremer, 2014; Shropshire and Bordenstein, 2016 for recent reviews)? Isolation barriers can be either pre-mating or post-mating. Pre-mating reproductive barriers may be driven by ecological or behavioral isolation. For instance, particular bacterial symbionts can confer novel traits (e.g., increased thermal tolerance or adaptation to new host plants), allowing their insect host to exploit new ecological niches (Montllor et al., 2002; Ferrari et al., 2004; Tsuchida et al., 2004). Niche expansions such as these can result in geographically or sympatrically isolated populations and, given enough time, lead to speciation. In addition, the microbiota has been implicated in behavioral changes related to mate choice, which may result in symbiont-driven behavioral isolation due to differences in courtship or mate discrimination (reviewed in Shropshire and Bordenstein, 2016). For example, *Wolbachia* plays a crucial role in driving pre-mating isolation between semispecies of the *Drosophila paulistorum* species complex (Miller et al., 2010). In addition, the gut microbiota influences kin recognition and mating investment in several *Drosophila* species (Lize et al., 2014). Specifically, both *D. bifasciata* and *D. melanogaster* are able to distinguish between mates that have a more similar or dissimilar microbiota to themselves (Sharon et al., 2010; Lize et al., 2014). This results in a tendency for assortative mating in *D. melanogaster* after feeding on different food sources (Sharon et al., 2010), although this behavior was replicated only in inbred laboratory lines (Najarro et al., 2015). Similarly, mate selection in scarab beetles is dependent upon immune competence that the females sense in the bacterial-derived male pheromones secretions (Leal, 1998; Vasanthakumar et al., 2008; Andert et al., 2010).

In contrast, post-mating reproductive barriers may be driven by genetic conflicts between host and microbes (i.e., *Wolbachia*) or a breakdown in holobiont complexes. In *Nasonia*, both types occur. *Wolbachia*-induced CI in this system is a pre-eminent case of symbiont-assisted isolation in which nearly complete CI levels (**Figure 2**) between the *Nasonia* species cause F1 hybrid lethality that is reversible by curing the *Wolbachia* infections. In other words, the interspecific F1 isolation is essentially undone with antibiotics that restore production of viable F1 hybrids (Breeuwer and Werren, 1990, 1995; Bordenstein et al., 2001; Raychoudhury et al., 2010a). The study system is notable in that it provided the opportunity to investigate whether *Wolbachia*-induced CI evolved early or late in the speciation process, i.e., before or after other interspecific pre- or post-mating barriers. While the “older” species pair, *N. vitripennis* and *N. giraulti*, diverged ∼1 million years ago and evolved other post-mating barriers such as high F2 hybrid mortality and abnormal courtship behavior (Breeuwer and Werren, 1995; Bordenstein et al., 2001), the very young species pair, *N. giraulti* and *N. longicornis*, diverged only ∼400,000 years ago and produce viable and fertile hybrids (Bordenstein et al., 2001). This observation indicates that *Wolbachia*-induced reproductive isolation via CI preceded the evolution of other post-mating barriers in the younger species pair, and therefore is the first major step in the speciation process (Bordenstein et al., 2001). The even younger species pair, *N. giraulti* and *N. oneida*, represents an interesting case in this context: *N. oneida* females show strong mate discrimination against *N. giraulti* males, but not vice versa, resulting in strong but incomplete and asymmetrical pre-mating isolation (Raychoudhury et al., 2010a). Moreover, the mate discrimination phenotype in *N. oneida* is recessive and lost in F1 hybrid females (Raychoudhury et al., 2010a). The impact of *Wolbachia* on this speciation event is unfortunately blurred by the recent *Wolbachia*-mitochondrial sweep from *N. giraulti* into *N. oneida* (Raychoudhury et al., 2009), which eliminated any *Wolbachia*-induced incompatibilities that may have existed previously. Therefore, pre-mating isolation is the only barrier currently preventing hybridisation between the two species.

An additional microbiota-mediated reproductive barrier has recently been uncovered in *Nasonia*, manifested as strong F2 hybrid lethality in interspecies crosses after curing of *Wolbachia* (Brucker and Bordenstein, 2013). Specifically, hybrid lethality between *Nasonia vitripennis* and *Nasonia giraulti* is reversed through removal of the *Nasonia* gut microbiota, and can be reinstated by inoculating germ-free hybrids with *Nasonia*-derived bacterial cultures (Brucker and Bordenstein, 2013). *Nasonia* hybrid lethality was also characterized by an altered gut microbiota in which a rare microbial genus became abundant in hybrids. The change in bacterial community structure was coupled with aberrant host immune gene expression (specifically differential regulation of serine proteases, antimicrobial peptides, and several signaling molecules from the IMD and Toll pathways) compared to the parental species (Brucker and Bordenstein, 2013). In this case, hybrid breakdown at the holobiont level led to severe mortality. This is the first study, to our knowledge, in which the microbial community contributes to hybrid mortality (**Figure 2**).

Changes in the microbiota could also result in other microbe-dependent reproductive barriers, similar to phenotypes observed in various animal systems, e.g., in terms of development time, behavior, and fecundity (Brucker and Bordenstein, 2012c). For example, species-specific cuticular hydrocarbons help in mate discrimination in *Nasonia* (Buellesbach et al., 2013), but the impact of the microbiota on mate-choice is unknown. The behavioral issues that arise in hybrids (Clark et al., 2010) may therefore have microbial underpinnings.

### Germ-Free and Gnotobiotic Rearing

A powerful aspect of the *Nasonia* model lies in the ability to selectively rear (or co-rear) *Nasonia* hosts in germ-free (Brucker and Bordenstein, 2012a; Shropshire et al., 2016), gnotobiotic (harboring a known, controlled microbiota) and transbiotic (harboring the microbiota of a different species) conditions. The ability to inoculate germ-free *Nasonia* larvae with monocultures or whole microbial communities will enable high precision studies that deconstruct the effects of specific microbial functions in *Nasonia*. These studies have the benefit of being implemented at any stage throughout the *Nasonia* developmental process, which is important to understand the assembly and regulation of the *Nasonia* microbiota. Specific host genes could also be knocked down to observe their direct effects on microbiota assembly and host-microbe interactions in the four different *Nasonia* species. With rising interest in utilizing CRISPR genome editing in *Nasonia* (Lynch, 2015), host gene addition and removal could soon be incorporated into the toolbox available for deciphering hologenomic interactions. With the unique environmental controls afforded by the *Nasonia* rearing system, there is ample opportunity to study microbiota influences on *Nasonia* development and fitness. With these tools at hand, the *Nasonia* model could also be used to experimentally test hologenomic evolution, for instance by exposing a wasp population to a selective pressure (e.g., an environmental stressor) and subsequently monitor (i) whether changes in the microbiota correlate with changes in host life history traits or behavior and (ii) whether this shift in the microbial community persists over multiple generations, as long as the selective pressure persists.

## Perspectives and Concluding Remarks

This review highlights the important contribution *Nasonia* has made regarding our understanding of the manifold roles of both heritable symbionts and the general microbiota on host fitness and evolution. By conducting full microbial community transplantation experiments using the established *Nasonia in vitro* rearing system, further studies can elucidate the genome-by-microbiome interactions that cause reproductive barriers in *Nasonia*. This, among many other advantages highlighted in this review, reinforces the *Nasonia* holobiont as a highly versatile and tractable model, allowing for a controlled approach to host-microbe analyses. An important direction for future research will be to investigate *Nasonia*-microbiota dynamics in more ecologically relevant settings and to test whether the patterns observed thus far in a small number of inbred laboratory lineages hold true under natural conditions. For instance, is phylosymbiosis evident in wild populations? Does the microbiota of a given *Nasonia* species change when parasitizing different fly species? Is there an exchange of microbes between different *Nasonia* species parasitizing the same fly host or does each species still ‘filter’ its characteristic microbiota from the common pool? Does the microbiota remain stable under different environmental conditions (e.g., when exposed to environmental stressors) or does it evolve in structure and function in a way that is malleable under given circumstances? It is beyond doubt that *Nasonia* will continue to provide valuable insights regarding the evolution of host-microbe interactions, especially as new tools for microbiome manipulation and functional interactions become available.

## Author Contributions

JD, EO, JS, SB, GH, and RB contributed ideas and concepts. JD, EO, GH, and RB wrote the paper. JD, EO, and RB made the figures. JD, EO, JS, SB, GH, and RB finalized the paper and figures.

## Conflict of Interest Statement

The authors declare that the research was conducted in the absence of any commercial or financial relationships that could be construed as a potential conflict of interest.
